# Stage‐ and histology‐specific sensitivity for the detection of lung cancer of the NELSON screening protocol—A modeling study

**DOI:** 10.1002/ijc.70045

**Published:** 2025-07-11

**Authors:** Koen de Nijs, Kevin ten Haaf, Juul Hubert, Dana Moldovanu, Carlijn M. van der Aalst, Harry J. M. Groen, Pim A. de Jong, Marjolein A. Heuvelmans, Matthijs Oudkerk, Harry J. de Koning

**Affiliations:** ^1^ Department of Public Health Erasmus MC—University Medical Center Rotterdam Rotterdam The Netherlands; ^2^ Faculty of Medical Sciences University of Groningen Groningen The Netherlands; ^3^ Department of Radiology University Medical Center Utrecht Utrecht The Netherlands; ^4^ Department of Epidemiology Rijksuniversiteit Groningen Groningen The Netherlands; ^5^ UMC Groningen–University Medical Center Groningen Institute for Diagnostic Accuracy Groningen The Netherlands; ^6^ Department of Pulmonary Medicine Amsterdam UMC Location Vrije Universiteit Amsterdam Amsterdam The Netherlands

**Keywords:** computed tomography, lung cancer, NELSON, nodule management, screening

## Abstract

The Dutch–Belgian lung cancer (LC) screening trial (Nederlands–Leuvens Longkanker Screenings Onderzoek [NELSON]) demonstrated low‐dose computed tomography (CT) reduces LC mortality by 24% among men. The NELSON protocol differed from previous trials in the eligibility criteria, the use of volume‐based nodule management, and increasing screening intervals. The early‐stage sensitivity of the protocol is pivotal in determining the optimal screening strategy, such as the interval and age range. The MIcrosimulation SCreening ANalysis‐Lung natural history model was used to reproduce LC incidence and mortality by detection method (clinical or screen‐detected), sex, histology, and stage in the NELSON trial based on individual‐level data. We evaluated screening effectiveness by stage and histology, accounting for population characteristics, trial design, and LC epidemiology. We find stage IA non‐small cell LC (NSCLC) sensitivity of 24.6% (other NSCLC) to 41.0% (adenocarcinoma) at baseline screening. At repeat screening rounds, we find this increased to 70.9% for stage IA adenocarcinoma. For stage IB, the sensitivity by histology ranges from 26.4% to 77.1%; for stage II, 39.6%–81.9%. Upon detection, the probability of LC mortality prevention is estimated at 83% for stage IA. The sensitivity for detecting early‐stage LC is found to depend on the histology of cancer and is increased for adenocarcinoma at repeat screenings. Despite a low rate of referral to follow‐up screening in the NELSON trial, early‐stage CT sensitivity and the probability of mortality prevention were similar to previous estimates from the National Lung cancer Screening Trial. Previously demonstrated screening effectiveness may be maintained when implementing new programs, while reducing unnecessary follow‐up when considering NELSON evidence.

## What's New?

The NELSON protocol has shown substantial promise in utilizing low‐dose computed tomography (CT) for lung cancer detection. However, its effectiveness across different disease stages remains poorly understood. Here, using NELSON data and the MISCAN (MIcrosimulation SCreening ANalysis)‐Lung model, the authors estimated NELSON‐specific CT sensitivity for early‐stage lung cancer detection. Detection sensitivity for stage IA adenocarcinoma was as high as 71% at repeat screens. Meanwhile, the estimated probability of preventing lung cancer mortality for stage IA was 83%. The findings indicate that the NELSON protocol can detect most early‐stage lung cancers, potentially helping to reduce unnecessary follow up.

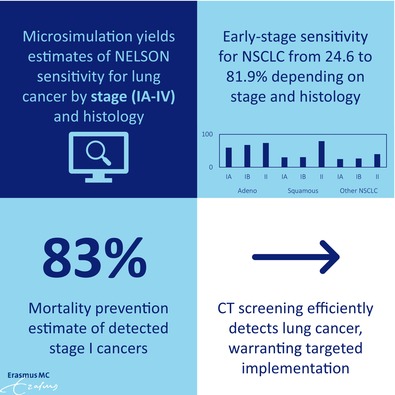

**Abbreviations**
CPD: cigarettes per day
CT: computed tomography
LC: lung cancer
MISCAN: MIcrosimulation SCreening ANalysis
NELSON: Nederlands–Leuvens Longkanker Screenings Onderzoek
NLST: National Lung cancer Screening Trial
PLCO: Prostate, Lung, Colorectal and Ovarian Screening Trial
TSCE: two‐stage clonal expansion
USPSTF: United States Preventive Services Task Force
VDT: volume doubling time

## 1 INTRODUCTION

Lung cancer (LC) is the leading cause of cancer‐related mortality worldwide, responsible for 1.8 million annual deaths.^1^ Clinical diagnosis of LC commonly takes place in an advanced, metastasized stage.^2^ To promote early‐stage detection, when curative treatment is more likely, high‐risk individuals may be screened with low‐dose computed tomography (CT). Screening with CT compared to chest radiography demonstrated a 20% reduction (8% in males^3^) in LC mortality in the National Lung cancer Screening Trial (NLST),^4^ and a 24% reduction (in males) relative to no screening in the Dutch–Belgian Randomized Lung Cancer Screening (Nederlands–Leuvens Longkanker Screenings Onderzoek [NELSON]) trial.^5^ Previously, we have shown the sensitivity of the NLST screening protocol by stage and histology of cancer.^6^ It is unknown what the stage‐specific sensitivity is for the NELSON protocol. The early‐stage sensitivity of screening is known to influence recommendations on the optimal screening strategy, such as the interval and age range.^7^

The NELSON trial employed a volume‐based nodule management protocol, which sought to reduce follow‐up procedures while maintaining screening effectiveness. Nodule classification protocols affect the overall CT sensitivity.^8^, ^9^, ^10^ The nodule classification protocol will affect which nodules are further evaluated.^11^ For example, the NLST referred nodules >4 mm to further follow‐up, for a 27.3% positivity rate. NELSON referred nodules >500 mm^3^, with 50–500 mm^3^ nodules scanned again at 3 months, for a total 2.6% positivity rate.^12^ Currently, the American College of Radiology recommends the diameter‐based Lung‐RADS protocol,^13^ while the British Thoracic Society advises the use of nodule volume to determine follow‐up, after the use of volume‐based nodule management in the UK Lung Cancer Screening Trial (UKLS).^14^, ^15^ Previous studies have compared the overall sensitivity between volume‐based and diameter‐based protocols, finding comparable sensitivity but higher specificity for volume‐based protocols^8^, ^16^, ^17^, ^18^ However, the sensitivity of the protocol by preclinical cancer stage (the Tumour, Node, Metastatis classification (TNM) stage a latent cancer would have if detected at the time of the screening) and histology have not yet been quantified.

To estimate the sensitivity of the protocol by stage of cancer, an estimate is required of the number of undetected cancers present in the screening population at the time of each screening event. To this end, one must account for the risk composition of the study population, the effects of previous screening rounds, and the projected rate of progression of the cancer throughout the trial period. To this end, natural history modeling of LC may be used, as previously demonstrated for a comparison of the Prostate, Lung, Colorectal and Ovarian Screening Trial (PLCO) and NLST trials.^6^, ^19^

We use the MIcrosimulation SCreening ANalysis (MISCAN)‐Lung model, previously calibrated to the NLST and PLCO, to replicate the NELSON trial. Specifically, we model how many cancers of each TNM stage (the assigned stage if they were detected at that moment) and histology are expected to be present undetected. This allows estimates of the CT sensitivity by stage and histology, by comparing the NELSON‐observed detected cancers by stage at each screening round to the estimated pool of undetected cancers. Often, sensitivity estimates of cancer screening tests compare the observed detected cancers to the number of cancers that are clinically detected in a subsequent fixed‐length period (empirical sensitivity).^16^, ^20^ This marks any clinically detected cancer shortly following the screening event as a false negative to the screening event, despite it being unknown whether it was already present. Conversely, it marks any cancers incident after the cut‐off period as true negatives, despite it being unknown whether the cancer was already visible at the screening event. LC natural history modeling accounts for this by estimating the pool of undetected cancers of each stage and histology that are prevalent at the time of the screening event, allowing point estimates of the CT sensitivity specific to the stage and histology of the cancer.

The purpose of this study is to investigate the screening effectiveness of the NELSON protocol, particularly the sensitivity of the CT for early‐stage cancers, and the rate at which early detection results in the prevention of a potential LC death. No such study of the sensitivity of CT and effectiveness of screening by histology and stage has yet been performed for the NELSON trial, despite European estimates of the benefits of screening leaning on the results from NELSON. NELSON‐specific estimates of screening effectiveness will be critical for extrapolations of trial results to population‐level implementations of LC screening, as proposed for EU Member States by the EU council and Australian Government.^21^, ^22^

## 2 METHODS

### 2.1 Data

The NELSON trial was a randomized controlled trial studying the effectiveness of LC screening with CT among high‐risk individuals. The nodule management and imaging protocol have been described previously.^23^ Participants, aged 50–74, were required to be currently smoking or have quit less than 10 years ago. Furthermore, eligibility required a minimum smoking exposure of either smoking >15 cigarettes per day (CPD) for >25 years or >10 CPD for >30 years. Recruitment started in 2003, with follow‐up on LC incidence and mortality up to 31 December, 2015, for a minimum of 10 years of follow‐up per participant. LC staging followed the seventh edition TNM staging system, contemporary to the trial.^24^ Our study is limited to Dutch participants (93.8% of NELSON participants), as for Belgian participants only summarized information on LC incidence and mortality was available. Cancers with an unknown stage had stage information imputed from patient and tumor characteristics, as further described in Supporting Information S1.

### 2.2 Model

The MISCAN‐Lung model has been described previously.^6^, ^19^ The model simulates individual life histories (epidemiological life events from birth until death), including smoking behavior, LC onset and mortality, and smoking‐behavior‐specific other‐cause mortality. The rates at which these events occur are calibrated such that the aggregate of many life histories matches population‐level smoking‐ and LC rates. The model can be used to extrapolate trial results to scenarios of population‐level screening interventions. In this capacity, it has previously informed 2013 and 2021 United States Preventive Services Task Force (USPSTF) guidelines, and has evaluated the cost‐effectiveness of LC screening in the United States, Canada, Switzerland, and Australia.^22^, ^25^, ^26^, ^27^, ^28^, ^29^, ^30^, ^31^, ^32^

**FIGURE 1:**
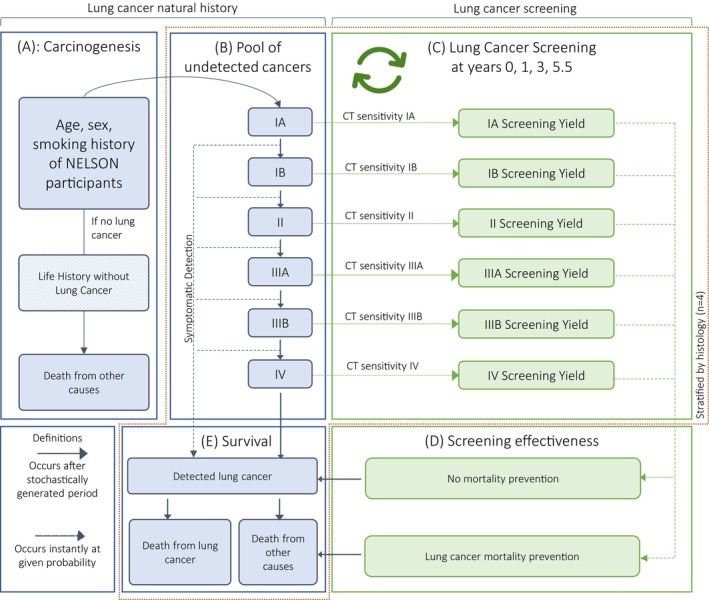
MIcrosimulation SCreening ANalysis (MISCAN) model structure: The structure of the MISCAN model. (A): The age of first lung cancer onset is calculated based on a dose–response model (two‐stage clonal expansion) of cigarettes per day over the life history. (B) Upon lung cancer onset, the cancer starts in stage IA. For each stage of cancer, a preclinical sojourn time is drawn. After the sojourn time, the cancer may be detected symptomatically at a given probability or progress to the next stage for which a new sojourn time will be drawn. (E) Upon clinical detection, a survival time is drawn. The individual may still die of other causes if a death from other causes is scheduled before the death from lung cancer. (C) In Nederlands–Leuvens Longkanker Screenings Onderzoek (NELSON), computed tomography (CT) screening was performed at baseline, Year 1, Year 3, and Year 5.5. If there is a cancer present, it may be detected with the CT sensitivity specific to the stage of the cancer at the time of the screening. If it is detected, the age of lung cancer death that would have been scheduled if it had been detected symptomatically can be prevented at a given probability, represented in panel (D). If the prevention is unsuccessful, the life history continues as if no screening had occurred. As noted in the figure, panels (B) and (C) depend on the histology of the cancer, which may be one of adenocarcinoma, squamous cell carcinoma, other non‐small cell lung cancer, or small cell lung cancer.

A diagram of the model is presented in Figure 1. First (panel A), lung carcinogenesis is modeled using the two‐stage clonal expansion model (TSCE), which estimates individual‐level risk of developing LC, as a function of sex, age, and history of smoking.^33^ After carcinogenesis occurs, the cancer spends a stochastically generated period, referred to as the preclinical sojourn time, in subsequent stages of cancer, moving from IA to IV, as shown in panel B. The Weibull distribution of sojourn times also includes very short sojourn times, which reflect cancers that may effectively skip past TNM stages. The distribution of the preclinical sojourn time is specific to the stage and histology of the cancer. A person may develop one of four possible cancer histologies: adenocarcinoma, squamous‐cell carcinoma, other non‐small cell LC, or small cell LC. The cancer may be detected clinically at the end of the sojourn time or progress undetected to the next stage. At clinical detection, a survival time is drawn from a Poisson stage‐ and histology‐specific survival curve, as shown in panel (E).^34^ Relative survival is calculated from the incidence of first primary LC onwards, such that recurrence and second primary LC are included in the relative survival. Individuals may die from LC or from other causes. Other‐cause mortality rates by birth cohort and sex from Statistics Netherlands are adjusted for the individual‐level smoking history.^35^

The model also includes the effect of CT screening attendance in the life history (panel C), where any preclinical LC may be screen‐detected with a sensitivity specific to the cancer stage and histology at the time of the screening.^6^ As shown in panel (D), when the screening successfully detects the cancer, the LC death may be prevented at a probability specific to the cancer stage. We refer to this as the mortality prevention probability. A 0% probability corresponds to screen‐detection having no effect on the LC mortality rate among the screen‐detected cases. A 100% probability indicates that all individuals with a screen‐detected LC in that stage are successfully cured or have treatment that postpones their cancer mortality in such a way that they die of other causes first. Together with the sensitivity of the CT for detecting LC, the mortality prevention probability yields the screening effectiveness, the probability that a CT screen effectively prevents LC mortality for a participant entering the screening with an as yet undetected cancer. A more elaborate description of the MISCAN model is presented in Supporting Information S1.

### 2.3 Calibration to the NELSON trial

The parameters of the MISCAN‐Lung model, such as sojourn time distributions and the sensitivity of screening tests, were previously calibrated to individual‐level data from the NLST and PLCO. This joint calibration allowed the estimation of chest x‐ray effectiveness (PLCO and NLST) and CT effectiveness (NLST).^6^ To allow for differences between the NELSON setting and that of the NLST, we recalibrate the MISCAN‐Lung model to individual‐level NELSON outcomes.

For a given set of model inputs, the model simulates the LC natural history of a NELSON‐like population to compare against observed NELSON outcomes. We use individual‐level data from NELSON baseline on sex, smoking history, and age to create a 200‐fold replication of the trial population in order to reduce simulation noise. The life history of each member of this population is simulated. Per the simulation structure shown in Figure 1, a LC onset is simulated, starting in stage IA, and sojourn times in subsequent stages of cancer are drawn. The LC may be detected either at NELSON screening rounds or through symptomatic detection between screening rounds or after the final screen. We also simulate smoking cessation, which may affect carcinogenesis, and other‐cause mortality from NELSON baseline onwards. Age‐ and sex‐specific patterns in attendance by screening round are applied.

The simulated outcomes, specifically the screening yield shown in panel C of Figure 1, are compared to observed outcomes to evaluate the fit of model parameters. The parameters can then be set to the values that best reproduce the observed NELSON screening yield. Specifically, the CT sensitivity by stage and histology can be set to detect the proportion of the pool of undetected LCs (panel B in Figure 1) that matches the observed screen‐detected cases in NELSON. To do so, a differential evolution algorithm^36^ is used to iteratively adjust the MISCAN‐Lung model toward a best‐fitting set of model inputs:

#### 2.3.1 Computed tomography sensitivity

A set of input parameters is calibrated to find the best‐fitting CT sensitivity by stage and histology for the NELSON trial, using a formula defined further in the methods, Supporting Information S1. Parameters governing the CT sensitivity by stage of cancer and by each histology are calibrated to get the stage‐ and histology‐specific sensitivity estimates. The NELSON nodule management protocol incorporated nodule growth in terms of volume doubling time (VDT) for the classification of nodules found in repeat screens,^5^, ^23^ which may affect sensitivity at repeat screens.^10^, ^16^ To quantify the benefit of this information on the CT sensitivity, we allow higher sensitivity for repeat screens than at baseline. A model run with baseline and repeat screening restricted to be equal was found to yield a poorer model fit to NELSON data. To obtain the average sensitivity by stage and histology for all rounds of the NELSON trial (baseline and repeat screens together), we divide the predicted detected cancers (true positives) by the estimated number of detectable cancers across all 4 screening rounds.

#### 2.3.2 Distribution of IA sojourn times

The detectability of cancer with a screening test is dependent both on the sensitivity of the test and the length of time for which the cancer is present in a presymptomatic, detectable state. We therefore estimate for the NELSON trial also the sojourn time of the earliest stage of cancer, stage IA. The sojourn times are drawn from a Weibull distribution with a mean and shape parameter, which determine, respectively, the average length and the variance of the time simulated cancers spend in stage IA, the most common stage of screen‐detected cancers. The mean and shape are calibrated to match the observed screening yield and interval cancer rate in NELSON screening rounds; for the same CT sensitivity, a high interval cancer rate, or a higher late‐stage yield at subsequent screening rounds, suggests short sojourn times or high variability, corresponding to many quickly growing cancers presenting. A low stage IA yield in repeat screening rounds corresponds to longer sojourn times, suggesting many cancers were already detectable in the baseline screen.

#### 2.3.3 Lung cancer mortality prevention

Panel D of Figure 1 shows the mechanism of the LC mortality prevention parameter. In the model, these report the probability that a cancer death in a no screening scenario is prevented if it is screen‐detected in the given stage. These probabilities were also estimated for the NELSON trial. We use the genetic algorithm^36^ to search for the mortality prevention rates that match the MISCAN‐Lung mortality rate among individuals with screen‐detected LC to the observed NELSON rates (per person‐year at risk for LC mortality, after screen‐detection).

#### 2.3.4 Country‐specific lung cancer epidemiology

Although the NELSON trial was reflective of the Dutch high‐risk population,^37^ our model should further be consistent with LC occurrence among the Dutch general population. To ensure validity of the model for population‐level LC incidence, we adjust parameters that may be heterogeneous for the Dutch epidemiological context of LC:

- Background‐ and smoking‐induced LC risk parameters of the TSCE carcinogenesis model.
- The histological distribution of LC.
- The probability of clinical detection in stages IA to IB, which may differ by health care system.^38^

To match the model outcomes to LC epidemiology in the absence of screening, we adjust these model parameters to LC incidence per the Netherlands Cancer Registry for the period 2000–2020. For the general population, we use rates of smoking prevalence and CPD obtained from the Dutch Health Survey (1989–2020).^39^

To evaluate the simulated outcomes from the MISCAN‐Lung model with a given set of parameters relative to observed NELSON outcomes, several calibration targets are used: LC incidence, stage distribution, and histology distribution by trial arm (control or CT‐screened) and method of detection (clinical or screen‐detected), as well as the mortality rate of individuals with screen‐detected cancers. A complete specification of calibration targets and inclusion therein of cancers with imputed stages is given in Supporting Information S1. The calibration of the sensitivity parameters in a disease natural history model does not allow the calculation of p‐values or confidence intervals as offered by regression models or common summary statistics. To offer an alternative, we explore the likelihood profile of the parameters around their maximum likelihood estimate, with which a set of feasible values can be generated. As also demonstrated in our previous study of the NLST, the calibrated parameters are raised and lowered incrementally to interpolate the points below and above the point estimate at which the likelihood increase crosses the 97.5 percentile of the chi‐squared distribution.^6^ This represents the interval for which, holding other parameters equal, the increase or decrease does not reject the null hypothesis of the new model fitting as good as the model at the point estimate, which we refer to as the range of feasible values. This approach is further explained in Supporting Information S1.

## 3 RESULTS

We used participant‐level data from the Dutch screening centers (Belgian participants excluded given data availability) of the NELSON trial consisting of 12,474 men and 2382 women, all of whom provided informed consent.^5^ Descriptive statistics of the trial population are given in Table S1, showing the population has a high smoking exposure. A 6% share of participants experience LC incidence within trial follow‐up. The distribution of cancers among TNM stages and histological subtypes is presented in Table S4. Among clinically detected cancers, stage IV cancers are most common, whereas stage IA cancers are the most prevalent among screen‐detected cancers. To facilitate estimates from the MISCAN‐Lung model, six records with invalid smoking history data (missing data for smoking duration or CPD) were excluded. The NELSON trial compared four screens with low‐dose CT at increasing intervals (1, 2, and 2.5 years between screens) to an unscreened control arm. Adherence to these rounds was 96.1%, 94.5%, 91.9%, and 70.5%, respectively, attributable to the final round requiring renewed consent as an addition to the initial trial design.^40^

### 3.1 Model validity

**FIGURE 2:**
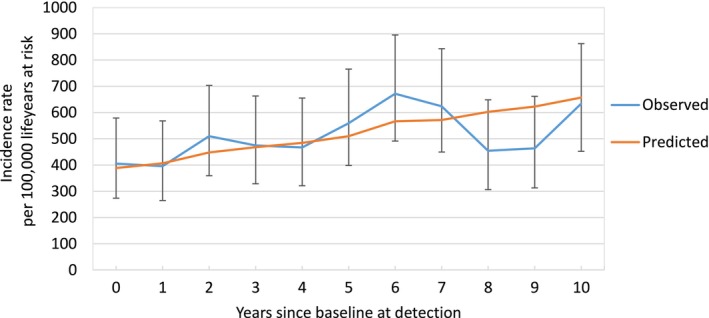
MIcrosimulation SCreening ANalysis (MISCAN)‐Lung predicted lung cancers in the Nederlands–Leuvens Longkanker Screenings Onderzoek (NELSON) control arm versus observed clinically detected cancers, by years since randomization: The MISCAN‐Lung‐simulated and observed lung cancer event rates in the control arm of the NELSON trial. Lung cancer events are aggregated to the year of incidence relative to baseline. MISCAN‐Lung estimates of lung cancer incidence in the NELSON control arm are generated per methods described in the model description in Supporting Information S1. We report 95% confidence intervals for the observed incidence levels based on a Poisson distribution.

**FIGURE 3:**
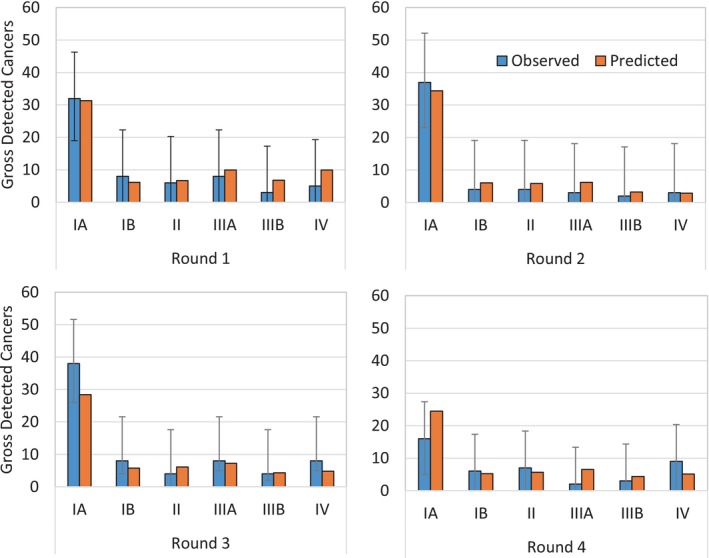
MIcrosimulation SCreening ANalysis (MISCAN)‐Lung predicted screen‐detected lung cancers in the Nederlands–Leuvens Longkanker Screenings Onderzoek (NELSON) trial vs observed cancers, by screening round and stage at detection: Gross number of MISCAN‐Lung‐predicted and observed screen‐detected lung cancers in the NELSON trial, by stage of cancer at the moment of detection and by screening round. MISCAN‐Lung estimates of lung cancer incidence in the NELSON control arm are generated per methods described in the model description in the Supporting Information S1. 95% confidence intervals assume a multinomial distribution of screens between negative screens and stage‐specific positive screens, per the method of Glaz and Sison.^41^

Figure S4 shows the predicted outcomes of MISCAN‐Lung relative to population‐level LC incidence. The projected LC rates for MISCAN‐Lung track the population‐level incidence rates. For the most common age group in the NELSON trial, those aged 55–59, the predicted incidence is 104.5 and 113.1 events per 100,000 lifeyears per MISCAN for females and males, respectively, relative to 104.6 (102.7–106.5) and 111.7 (109.7–113.7) observed. The fit of the MISCAN‐Lung model to the NELSON control arm is shown in Figure 2. For each period since randomization, we find predicted LC incidence to be within the 95% Poisson confidence intervals of the observed incidence. The close fits indicate that the MISCAN‐Lung model is able to predict LC risk of NELSON control‐arm participants from their reported age, sex, and smoking history. The parameters associated with LC incidence in the absence of screening can be found in Table S2. Figure 3 shows the expected number of screen‐detected cancers by stage of cancer and screening round, after calibrating the parameters of the model to the NELSON calibration targets. The screening yield by stage of cancer and screening round is within the 95% confidence interval of the observed yields in NELSON for each value presented in Figure 3. Figure S2 demonstrates the fit of the MISCAN‐Lung model to the rates of mortality among screen‐detected cases in the NELSON trial, which are reproduced within 95% confidence intervals for each stage of cancer upon detection. Additionally, Figure S3 shows the MISCAN predicted interval cancer rate by stage and round for NELSON, which we also found closely matches the observed rates.

### 3.2 Parameter estimates

Recalibration of the MISCAN‐Lung model to the results of the NELSON trial yields novel estimates of the CT sensitivity by stage and histology (the rates in panel C of Figure 1), a longer estimate of the sojourn time of stage IA adenocarcinoma than previous model estimates from the NLST suggested (the length of time spent in cell IA in panel B of Figure 1), and new estimates of the mortality prevention probability (per panel E of Figure 1). For histological types other than adenocarcinoma, we do not find stage IA cancer to have a longer sojourn time than previous results suggested. We present these parameters together to previous estimates from the NLST to show their relative change from previous evidence.

#### 3.2.1 CT Sensitivity

The estimated CT sensitivities by stage and histology of LC are given in Table 1. Per our estimates for the NELSON study, average CT sensitivity across all screening rounds for the detection of adenocarcinoma is 59% for stage IA in NELSON. We estimate the NELSON sensitivities separately by baseline (round 1) and repeat screening (rounds 2+). We find that the CT sensitivity is higher in repeat screenings for adenocarcinoma, but find no difference in sensitivity (magnitude difference below 1% point) of the repeat screen relative to the baseline screen for other histological types. In the final model, these are therefore set equal to baseline sensitivity. The separate estimates for baseline and repeat screens suggest a sensitivity in NELSON at baseline of 41% for stage IA and 71% for repeat screens. For squamous cell carcinoma, early‐stage sensitivity is found to range from 31.0% for stage IA to 79.3% for stage II. For other NSCLC, this is 24.6% (IA) to 39.6% (II). Late‐stage sensitivity is high across each NSCLC histology, from 67.5% for stage IIIA other NSCLC to 99.9% for stage IV adenocarcinoma and squamous cell carcinoma. For small cell LC, no early‐stage cancers were detected in the NELSON trial. For late‐stage small‐cell lung cancer (SCLC), we estimate a 78.7% (stage IIIA) to 99.9% sensitivity (stage IV). A likelihood profile analysis of the uncertainty of the parameter estimates is presented in Supporting Information S1. We find a range of feasible values of (−5.8%, +5.9%) for the CT sensitivity estimates.

**TABLE 1:** MIcrosimulation SCreening ANalysis (MISCAN) model‐estimated computed tomography sensitivity in the Nederlands–Leuvens Longkanker Screenings Onderzoek (NELSON) trial.

	NELSON all screen average (%)^a^	NELSON baseline screen (%)	NELSON repeat screen (%)	NLST all screens (%)
Adenocarcinoma				
IA	59.4	41.0	70.9	56.6
IB	67.5	49.0	77.1	64.1
II	73.7	56.3	81.9	64.5
IIIA	99.3	99.3	99.3	75.9
IIIB	99.7	99.5	99.8	80.2
IV	100.0	99.9	100.0	98.9
Squamous cell carcinoma^b^				
IA	30.1	30.1	30.1	31.0
IB	30.4	30.4	30.4	38.0
II	79.3	79.3	79.3	39.2
IIIA	96.7	96.7	96.7	69.7
IIIB	99.3	99.3	99.3	79.4
IV	99.9	99.9	99.9	97.7
Other NSCLC^b^				
IA	24.6	24.6	24.6	20.8
IB	26.4	26.4	26.4	24.8
II	39.6	39.6	39.6	24.8
IIIA	67.5	67.5	67.5	60.4
IIIB	89.8	89.8	89.8	68.3
IV	99.2	99.2	99.2	95.7
Small‐cell lung cancer^b^				
IA^c^	8.8	8.8	8.8	8.8
IB^c^	10.3	10.3	10.3	10.3
II^c^	11.2	11.2	11.2	11.2
IIIA	78.7	78.7	78.7	41.6
IIIB	96.8	96.8	96.8	87.1
IV	99.9	99.9	99.9	99.4

*Note*: Table 1 reports estimates of the sensitivity of a computed tomography scan for detecting a lung cancer of a given stage and histology. Estimates from a previous study of lung cancer detectability in the NLST are reported, as well as novel estimates based on outcomes from the NELSON trial. Estimates are derived from microsimulation with the MISCAN‐Lung model, which finds the best‐fitting sensitivity to reproduce the NELSON screening yield, taking into account the smoking histories of NELSON participants and the lung cancer epidemiology of the Netherlands.

Abbreviation: NSLT, National Lung cancer Screening Trial.

^a^ The NELSON all‐screen sensitivity is calculated by dividing the predicted number of detected cancers by the total number of detectable cancers in the model across all four screening rounds.

^b^ Estimated $\beta_{h}=0$, that is, no differences found between repeat and baseline screen sensitivity for squamous‐cell, small‐cell and other NSCLC cancers.

^c^ Not recalibrated for the MISCAN‐Lung NELSON calibration, estimates from the NSLT are shown for reference; the stage‐histology combination was not observed in NELSON.

#### 3.2.2 Preclinical sojourn time

To account for the possibility that the cancer is detectable for a longer period than previous evidence suggested, we also calibrate the time spent in stage IA LC, which we find to be increased in NELSON only for adenocarcinoma relative to our previous estimates. For other histologies, the estimates for the sojourn times of stage IA were found to be negligibly close to previous estimates and set to the initial value in the final model. The estimates are reported in Table 2. We find a mean time spent in IA adenocarcinoma of 3.6 years for men (1.8 per NLST estimates) and 4.8 years for women (2.4 per NLST estimates).

**TABLE 2:** MIcrosimulation SCreening ANalysis (MISCAN)‐Lung adenocarcinoma sojourn time estimates for the Nederlands–Leuvens Longkanker Screenings Onderzoek (NELSON) trial and the National Lung cancer Screening Trial (NLST).

Histology	Stage	Sex	NELSON sojourn time mean	NELSON sojourn time shape^a^	NLST sojourn time mean^6^	NLST sojourn time shape^6^
Adenocarcinoma	IA	Male	3.56	0.35	1.82	1.44
Adenocarcinoma	IA	Female	4.77	0.35	2.43	1.44

^a^ Sojourn times are drawn from a Weibull distribution, which takes both mean and shape parameters. MISCAN‐Lung estimates the time spent in stage IA adenocarcinoma per outcomes from the NELSON trial, as compared to previous estimates for the NLST.^6^ For other histological types, we find no difference in the sojourn time estimate; their values and those for other TNM stages are reported in Table S6.

#### 3.2.3 Probability of mortality prevention

**FIGURE 4:**
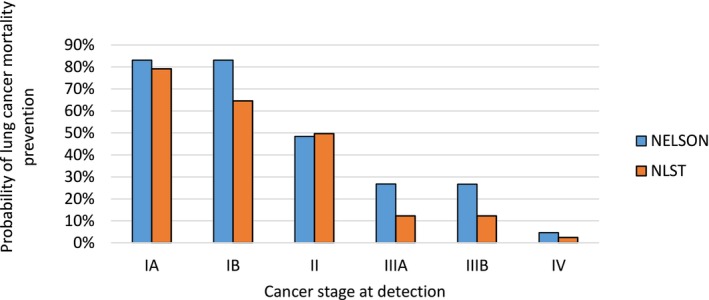
Estimated screening effectiveness (probability of lung cancer mortality prevention) by trial and stage of cancer: MIcrosimulation SCreening ANalysis‐Lung estimated probabilities of a screen‐detected lung cancer resulting in mortality prevention in the Nederlands–Leuvens Longkanker Screenings Onderzoek (NELSON) trial, as compared to earlier estimates for the National Lung cancer Screening Trial (NLST).^6^ Parameter estimates are based only on cancers with an observed stage (not imputed).

Figure 4 shows the estimated probabilities of successful prevention of LC mortality after screen‐detection. We find that stage IA and IB cancers are associated with an expected 83% probability of LC mortality prevention. Comparing this to previous estimates from the NLST, we find the NELSON rates slightly higher across stages.

## 4 DISCUSSION

In this study, we evaluated the stage‐ and histology‐specific sensitivity for the detection of LC by CT in the NELSON trial. The MISCAN Lung microsimulation model was recalibrated to NELSON outcomes. We found the MISCAN‐Lung model to be able to accurately replicate the NELSON trial results and population‐level LC incidence for the Netherlands. We found early‐stage sensitivity for NSCLC to depend on the histology and round of screening. Stage IA sensitivity for other NSCLC was 20.8%, whereas sensitivity for stage II adenocarcinoma was estimated as 81.9% for repeat screens. Additionally, we estimated the average stage IA adenocarcinoma to be present for longer than previously thought,^6^ by 1.7 years for men and 2.3 years for women.

A higher CT sensitivity at repeat screens, particularly for stage IA adenocarcinoma, which is the most common screen‐detected stage and histology combination, is consistent with the potential added benefit of incorporating nodule volume growth in the nodule classification. Malignancies may be identified that would not be further investigated based on diameter or volume, but warrant biopsy based on their rate of growth. For this, repeat attendance of CT screening is important to track existing nodules over time. Additionally, it has been shown that the NELSON protocol is associated with fewer total referrals for follow‐up examinations.^16^ This benefits sparse use of resources and may reduce patient anxiety surrounding unnecessary follow‐up. But to preserve the value of screening, it should not be associated with lower rates of detection.

In our previous study of CT sensitivity for the NLST^6^, where a protocol was used with higher rates of follow‐up, we found similar estimates of early‐stage sensitivity. For stage IA adenocarcinoma, we estimated 56.6% sensitivity in the NLST. In the current study, this was 41.0% for baseline screens and 70.9% for repeat screens; 59.4% average across the screening rounds. It should be noted that this is after recalibration of the natural history of disease to the NELSON setting, including longer estimates for the sojourn time of stage IA adenocarcinoma. This result may also explain the higher relative benefit of female NELSON participants (although not statistically significant), who are more likely to present with adenocarcinoma than other histologies.

Finally, we also estimate the probability of mortality prevention by stage. An individual detected with stage IA cancer had an estimated 79% probability of lifetime LC mortality prevention in the NLST, which we found to be slightly higher at 83% in NELSON. Although a favorable estimate for the benefits of LC screening, this cannot be directly attributed to any one determinant of screening effectiveness. Numerous differences between the two trials existed, such as the population characteristics, the clinical care setting, and the use of chest x‐ray in the control arm of the NLST population. The MISCAN model was adjusted to the LC incidence, survival, and stage distribution of the Dutch population to be representative of the NELSON trial and for any future modeling of population‐level harms and benefits. The mortality benefit is therefore estimated relative to the average outcomes of LC patients in the NELSON control arm the general population.

Overall, we find similar estimates of screening effectiveness in NELSON to our previous estimates from the NLST. This may lead to considerations of which protocol of screening is optimal for population‐level implementation. Similar to the NLST, but with higher diameter thresholds of nodule follow‐up, the U.S. Lung Imaging Reporting and Data System (Lung‐RADS) guidelines for CT screening make use of nodule diameter for scheduling follow‐up screening. British Thoracic Society guidelines have adapted the volume‐based protocol employed in the NELSON trial.^15^, ^42^ The NELSON protocol was associated with a higher specificity, referring a lower proportion of participants to further examination.^8^ Our findings suggest that the nodule management protocol does not come at a cost of decreased LC detectability. Depending on the histological type and stage, some CT sensitivity estimates are higher than previously estimated. These results may inform nodule management protocols, suggesting a benefit of volume‐based protocols in test characteristics for the detection of malignant nodules. Our model recalibration to NELSON also finds the most prevalent screen‐detected cancer, stage IA adenocarcinoma, to have a preclinical sojourn time longer than previously thought. A longer preclinical sojourn time presents opportunities for efficient screening with longer screening intervals than the annual screening maintained in the NLST. These results may be further informed by outcomes from the ongoing 4‐IN‐THE‐LUNG‐RUN trial,^43^ which compares biennial LC screening to annual screening for individuals with a negative baseline screen.

We do note a few limitations. The NELSON trial had fewer participants than the NLST. Consequently, our estimates for the difference in sensitivity for less prevalent histological types have a degree of uncertainty, as presented by the likelihood profiling analysis. To get a robust estimate of the CT sensitivity, the natural history model needs to match the epidemiology of the population in question. We replicate the natural history of LC for NELSON participants as closely as possible, controlling for characteristics of the study population and the trial design. The external validity of our results then depends on the validity of the NELSON population for other screening‐eligible individuals in the Netherlands and abroad. NELSON participants have been shown to be similar at baseline to non‐participating screening‐eligible individuals.^37^ Additionally, we show that MISCAN‐Lung can accurately reproduce LC epidemiology for the general Dutch population during the timeframe of the NELSON trial. However, more evidence may be needed to further validate the sensitivity of CT for the detection of LC, particularly for population‐level programs or settings abroad. Additionally, to overcome the data limitations from the NELSON study, more data is needed to inform estimates for the less common stage and histology combinations. For example, we are not able to further stratify our estimates, for example, for subsolid and solid lung nodules. Further changes to nodule follow‐up protocols relative to the NELSON protocol, as well as improved imaging or the integration of AI and biomarkers,^44^, ^45^, ^46^ may also influence the (in)sensitivity of a CT protocol for the detection of LC; we offer a snapshot of the sensitivity of a continually evolving method of early cancer detection.

Multiple avenues for future research are apparent. Current estimates of the cost‐effectiveness of population‐wide implementation of LC screening do not account for the increased sensitivity for stage IA cancers seen in repeat screens of the NELSON trial.^27^, ^28^, ^29^, ^30^ New cost‐effectiveness studies are needed to ascertain whether these results affect optimal screening strategies. Increased rates of detection may allow longer screening intervals; lower false‐negative rates increase the confidence that an individual is LC free in the foreseeable future. Moreover, the longer preclinical sojourn time estimated for IA adenocarcinoma, the most common screen‐detected cancer, should inform new long‐term estimates of overdiagnosis in LC, for which modeling over the lifetime is essential.^47^, ^48^

To conclude, this analysis shows the probability of detection and the probability of mortality prevention are estimated to be more favorable in the NELSON setting than previous evidence suggested, a result which impacts clinicians and policy makers considering screening implementation and protocol design. A protocol based on volume‐based nodule management, as employed in NELSON, is known to refer fewer screening participants to follow‐up screening compared to diameter‐based protocols. Here, we show this does not come at a cost of lower LC detectability. We expect this contributed to the difference in mortality reduction between NELSON and the NLST, particularly given the lower overall rate of follow‐up examinations in the NELSON trial.

## AUTHOR CONTRIBUTIONS

**Koen de Nijs:** Conceptualization; methodology; software; validation; formal analysis; writing – original draft; writing – review and editing; data curation; visualization. **Kevin ten Haaf:** Conceptualization; methodology; software; investigation; writing – original draft; supervision; project administration; data curation. **Juul Hubert:** Writing – review and editing; investigation. **Dana Moldovanu:** Writing – review and editing; investigation. **Carlijn M. van der Aalst:** Investigation; writing – review and editing; data curation. **Harry J. M. Groen:** Writing – review and editing; investigation. **Pim A. de Jong:** Writing – review and editing; investigation. **Marjolein A. Heuvelmans:** Writing – review and editing; investigation. **Matthijs Oudkerk:** Writing – review and editing; investigation; funding acquisition. **Harry J. de Koning:** Conceptualization; writing – review and editing; supervision; funding acquisition; project administration; investigation.

## FUNDING INFORMATION

This work was supported by grants from the European Union Horizon 2020, and a VENI grant from the Dutch Research Council/Netherlands Organization of Health Research (ZonMW) (grant no. 09150161910060).

## CONFLICT OF INTEREST STATEMENT

Koen de Nijs reports grants from the NIH and the University of Zurich. Kevin ten Haaf reports grants for the submitted work from Dutch Research Council/Netherlands Organization of Health Research (ZonMW) Grant number 09150161910060, European Union (Horizon 2020) Grant agreement ID: 848294. Outside of the submitted work, he reports grants from NIH (1U01CA199284‐01), University of Zurich, Cancer Research UK (C35238/A26388), Cancer Australia, Medical Services Advisory Committee Australia, and the Open Mind Call Convergence (Erasmus University Medical Center Rotterdam, Erasmus University Rotterdam, Technical University of Delft). Payment for lectures to his institution: CHUV, Johnson & Johnson, DKFZ. Traveling support: CHUV, Rescue Lung, IASLC, DKFZ. Juul Hubert and Dana Moldovanu report a grant from the European Commission, paid to their institutions. Carlijn M. van der Aalst reports grants from the European Union, ZonMW, and the NIH, and committee membership at WHO‐IARC, B3Care. Pim A. de Jong reports consulting fees from Vifor Pharma. Marjolein A. Heuvelmans, Harry J. de Koning, Harry J.M. Groen, and Matthijs Oudkerk report no disclosures.

## ETHICS STATEMENT

The NELSON trial was approved by the Dutch Ministry of Health and the respective medical ethics committee at each participating center. All participants issued written informed consent.

## Supporting information


**Data S1.** Supporting Information.

## Data Availability

For the availability of NELSON patient‐level data, we refer to the data sharing agreement issued together with the original publication of NELSON trial results.^5^ MISCAN model results beyond those presented and the data that support the findings of this study are available from the corresponding author upon reasonable request.
