# Supplementary material for: Stage‐ and histology‐specific sensitivity for the detection of lung cancer of the NELSON screening protocol—A modeling study
Source: Int J Cancer. 2025 Jul 11;157(11):2248–58. doi: 10.1002/ijc.70045 (PMC12496006; doi:10.1002/ijc.70045)
Supplement: Supplementary file 1 — Data S1. Supporting Information. [file IJC-157-2248-s001.pdf]

Supplement to "Stage- and histology specific sensitivity for the detection of lung cancer of the NELSON lung cancer screening protocol"

Koen de Nijs, Kevin ten Haaf, Juul Hubert, Dana Moldovanu, Carlijn M. van der Aalst, Harry J.M. Groen, Pim A. de Jong, Marjolein A. Heuvelmans, Matthijs Oudkerk, Harry J. de Koning

Contents

Model Description 2
MISCAN-Lung 2
Population-level simulation 4
Simulation of NELSON 6
Calibration Targets 7
Estimating the range of feasible values 8
Supplementary Tables 11
Supplementary Figures 23
References 28

Model Description
MISCAN-Lung
The MIcrosimulation SCreening ANalysis (MISCAN) Lung model is a stochastic, microsimulation model.[1] In brief, the model simulates individual life histories in the considered population from birth until death, in the presence or absence of a screening program. Through comparing the life histories in the presence of screening with the corresponding life histories in the absence of screening, MISCAN-Lung can estimate the effectiveness and costs of screening scenarios. MISCAN-Lung was initially calibrated to individual-level data from the National Lung Screening Trial (NLST) and the Prostate, Lung, Colorectal, and Ovarian Cancer Screening Trial (PLCO).[2] [3] Per the methods outlined in this supplement, the MISCAN Lung model is recalibrated to individual-level outcomes from the (NELSON) trial. The following sections provide additional details on the MISCAN-Lung model, as well as the methods of recalibration.

General model structure

MISCAN-Lung is a semi-Markov model, which generates durations for a sequence of states. Individuals are simulated one at a time, which allows future state transitions to depend on past transitions giving the model a “memory”. MISCAN-Lung simulates sequences of events by drawing from distributions of probabilities/durations, which makes the results of the model subject to random variation.

MISCAN-Lung consists of several modules: a demography/smoking history generator module, a smoking-dose response module for lung carcinogenesis, a natural history module and a screening module.

Demography/smoking history generator module

First, birth-tables, representative for the population under consideration, are used to draw a date of birth for each simulated individual. Then, a smoking history is generated. Age, sex and five-year birth-cohort specific smoking initiation probabilities, representative for the population under consideration, are used to determine whether an individual initiates smoking and at what age. Upon smoking initiation, persons enter one of five smoking intensity categories. Average cigarettes smoked per day for each year in the life history are generated for each individual that initiates smoking, accounting for their birth cohort, sex and assigned category of smoking intensity. If an individual initiates smoking, age, gender and cohort specific smoking cessation probabilities are used to determine whether an individual ceases smoking and the age of smoking cessation.

Smoking related mortality

Upon generating a person’s smoking history, the age of death from causes other than lung cancer is generated, using mortality probabilities based on the person’s smoking history (smoking duration, smoking intensity category and average number of cigarettes per day, smoking status and years since cessation, if applicable), year of birth, age and sex. The maximum age an individual can achieve in MISCAN-Lung is exactly 100 years.

Smoking-dose response module for lung carcinogenesis

The smoking-dose response module allows modeling lung carcinogenesis as a function of a person’s age, gender and smoking history. MISCAN-lung utilizes the two-stage clonal expansion model (TSCE) as described by Heidenreich et al., as its smoking-dose response module (which estimates a person's risk of lung cancer, as a function of age and smoking history).[4] The parameters of the TSCE were obtained through calibration to the Nurses’ Health Study and the Health Professionals Follow-up Study [5]. However, the sex-specific parameters for malignant transformation were recalibrated to data from the National Lung Screening Trial (NLST), the Prostate, Lung, Colorectal and Ovarian cancer screening trial (PLCO) and the Surveillance, Epidemiology, and End Results (SEER) Program. [2] [3]

Natural history module

Lung cancers are assumed to progress sequentially through stages IA to IV, as shown in Supplementary Figure 5. The probability that a lung cancer progresses to a more advanced preclinical stage or is diagnosed clinically (e.g., diagnosed due to symptoms) is modelled by histology and stage. Histology is assigned at lung cancer onset, per rates reported in Supplementary Table 3. The transition probabilities by histology are reported in Supplementary Table 5. After clinical diagnosis, lung cancer survival is simulated using sex-, stage-, and histology specific survival estimates, obtained from the Dutch Cancer Registry for all individuals with lung cancer incidence 2000-2012, considered contemporary to the NELSON trial. The cumulative survival at 1, 2, 5 and 10 years is given in Supplementary Table 7. In the MISCAN-Lung simulation, when both a time-to-death from lung cancer and an age of death from other causes has been drawn, the death is set to the earliest simulated date of death (either due to lung cancer or other causes).

The preclinical durations (in the absence of screening), by histology, stage and gender were calibrated to the rates of screen-detected and interval cancers observed in the NLST and PLCO trials using individual-level data.[2] The preclinical durations (in the absence of screening) are drawn from Weibull distributions.

Screening module

Screening may detect cancers in each of the preclinical screen-detectable states, depending on the sensitivity of the screening test for the specific histology and preclinical stage. Upon detection of lung cancer by screening, a person's life history may be altered. Detection by screening may prevent the lung cancer death, allowing the patient to resume their normal (lung cancer free) life history. The probability of lung cancer mortality prevention differs by the stage at detection. Negative effects of screening, such as overdiagnosis of lung cancer (described subsequently), are also modelled.

Population-level simulation

To calibrate the MISCAN-Lung model inputs to the NELSON setting, we evaluated the model's potential to replicate Dutch lung cancer incidence. This ensures that model is representative of broader lung cancer epidemiology in the Netherlands.

We simulate lung cancer outcomes for the years 2000-2020 for cohorts 1935 to 1979. Smoking initiation, cessation and smoking-related other-cause mortality are calibrated to cohort life tables from the bureau of statistics and smoking prevalence per the Dutch Health Survey (1989-2020). Microdata from the Dutch Health Survey informs our estimates of the quintiles of smoking intensity in cigarettes per day, by sex and 5-year cohort. National tobacco sales contemporary to the Dutch Health Survey are

used to evaluate quantity underreporting by health survey respondents, yielding an estimate of 21% underreporting, consistent with previous estimates for other contexts[6-8]. Together, these estimates of smoking behavior are used to inform the Smoking History Generator component of the MISCAN-Lung model. The fit of the smoking history generator to observed current smoking prevalence over time by cohort are given in Supplementary Figures 6 and 7.

For a given set of model inputs, 10,000,000 life histories are simulated. The sizes of the individual cohorts constituting this population are set to comply to 2010 cohort sizes per Statistics Netherlands. For each simulated individual, a smoking history and smoking related other-cause date of death are drawn. Depending on the smoking history, a lung cancer natural history may be established. Lung cancer outcomes are noted for the years 2000-2020 to compare to population-level calibration targets of lung cancer incidence. Simulated and recorded outcomes include lung cancer incidence and mortality by histology (Adenocarcinoma, Squamous Cell Carcinoma, other non-small cell lung cancer, and small cell lung cancer) and stage of cancer at incidence (stages IA, IB, II, IIIA, IIIB and IV). The fit of the MISCAN model to lung cancer incidence by age in the Netherlands is shown in Supplementary Figure 1.

Simulation of NELSON

We simulate the life histories of NELSON participants from the start of their participation in the trial, and compare simulated outcomes to observed NELSON outcomes. For each participant, we use the MISCAN-lung model to simulate their natural history for 200 repetitions. To facilitate the simulation of individual-specific lung cancer incidence, we infer smoking histories from the responses to the smoking status questions at NELSON intake. For each participant the number of cigarettes per day (CPD), the age of initiation, and the total duration of smoking (if not smoking at intake) was used to model their smoking history.

Participants report their estimated number of cigarettes per day in discrete categories. To assign CPD values, we assume midpoints for each CPD category, reported in Supplementary Table 8. Similarly, for the ages of smoking initiation and cessation (years since cessation), midpoints of a self-reported category were assumed, also reported in Supplementary Table 8.

Each participant is assigned a smoking history. We assume that participants start smoking at the midpoint of the reported age of initiation bracket. The number of cigarettes per day is assumed to be the midpoint of the reported CPD bracket. The cigarettes per day over the lifetime are scaled to a smoking intensity curve per the Netherlands-specific MISCAN-Smoking History Generator. The scaling is performed such that the average CPD smoked over the person's smoking history from smoking initiation up to baseline concurs with the self-reported value at randomization. If the participant has stopped smoking, we assume that they smoked for a total number of years equal to the midpoint of their reported smoking duration years. Otherwise, the participant is assumed to quit smoking after baseline at rates consistent with the MISCAN-Smoking History Generator cessation rates particular to their sex and birth cohort.

Control Group Simulation

We simulate lung cancer natural histories for each participant in the control group. Other-cause-mortality is also simulated from the age of randomization onwards, using the MISCAN-Lung mortality rates specific to the smoking history, the sex and the birth cohort of the individual.

Screening Group Simulation

For the screening group, we also simulate invitation and attendance at lung cancer screening with low-dose CT. We assume that each individual attends the screening per the round-specific attendance rates observed in the NELSON trial of their age-group at baseline and sex. The timing of the CT screening round is rounded to the nearest 6-month interval since randomization. This facilitates large-scale simulation of the lung cancer screening rounds, whilst still approximating the individual-

specific timing of screening rounds. For each participant, age at randomization is only given in integer years. We therefore assume that the screening round starts halfway through that age year, since all participants with age x are expected to be uniformly distributed on the age interval [x, x+1).

Calibration Targets

The model was fit to several calibration targets from the NELSON control and screening arms, as well as population-level lung cancer outcomes. The calibration targets are stratified to ensure model validity across ages categories, sex, and cancer types. To create these categories, the NELSON data is stratified by: sex, age group (ages 50-54, 55-59, 60-64, 65-69 and 70-74 at randomization), trial arm (computed tomography/control arm), screening round (round 1, 2, 3 or 4) and year of diagnosis (1-10 years after randomization). Incident cancers are categorized by histology and stage of cancer at detection. This stratification will be used to calibrate the model to the calibration targets reported in Supplementary Table 9.

For 21 and 27 cancers in the NELSON control- and screening arms, respectively, the stage of cancer was not known. As not to underestimate the total screening yield per the NELSON protocol, the stage of these cancers was imputed for the estimation of the CT sensitivity, clinical detection rates, sojourn time and histology distribution. Cancer stage was imputed by multinomial logistic regression, as implemented in the Python autoimpute package, from patient characteristics (packyears, smoking status, sex, age at incidence, all-cause survival) and tumour histology.

The mortality prevention parameters by stage were estimated both with and without the imputed cancers to observe sensitivity to their inclusion. Survival of the cancers imputed to stage IV was better than cancers observed as stage IV, leading to a higher value of the stage IV mortality prevention than was deemed clinically realistic (17.3%). We therefore present the results of the calibration of the mortality prevention parameter without the imputed cancers in Figure 4 of the main manuscript, with an estimated mortality prevention of 4.6% for stage 4 screen-detected cancers.

Model calibration is performed with the differential evolution algorithm, per the implementation in the python package Scipy. Calibration is performed up to a tolerance of 0.00001, with a population size of 3 times the number of parameters calibrated. The parameters recalibrated to the NELSON trial are reported in Supplementary Table 10. Parameters were restricted to prevent evaluations of clinically implausible values (as shown in Supplementary Table 10).

Calibration of the CT sensitivity

A set of input parameters are calibrated to find the best fitting CT sensitivity by stage and histology for the NELSON trial. Specifically, we model CT sensitivity as:

$$P(True\ Positive\ |s,h) = f((\sum_{i=1A}^{s} \alpha_{i,h}) + \beta_h I_{screen\ 2+}),$$

where $f(x) = \frac{e^x}{1+e^x}$ is the logistic function, which restricts sensitivity estimates to be between 0-100%. s is the stage of the cancer, and h the histology. $\alpha_{i,h}$, being summed of all previous stages, reflects the increment in CT sensitivity compared to the previous stage (e.g., the sensitivity of IB compared to IA). $I_{screen\ 2+}$ is an indicator function that takes the value of 1 for any repeat screening. We allow higher sensitivity for repeat screens than at baseline to account for any contribution of volume doubling time to the sensitivity of the CT scan. A model run with baseline and repeat screening restricted to be equal was found to yield a poorer model fit to NELSON data. β_h captures the increment in sensitivity for repeat screens. We restrict $\alpha_{i,h} > 0$ for any $i > IA$, and $\beta_h > 0$, such that sensitivity increases by stage, and is minimally higher for repeat screens than baseline screens. For example, for the detection of stage II adenocarcinoma at a repeat screen, the sensitivity is given by:

$$P(True\ Positive\ |\ stage\ II\ Adenocarcinoma)$$
$$= f(\alpha_{IA\ Adeno} + \alpha_{IB\ Adeno} + \alpha_{II\ Adeno} + \beta_{Adeno}),$$

To obtain the average sensitivity by stage and histology for all rounds of the NELSON trial (baseline and repeat screens together), we divide the predicted detected cancers (true positives) by the estimated number of detectable cancers across all 4 screening rounds.

Estimating the range of feasible values

Descriptive statistics of a data sample, as well as regression estimates, allow for the calculation of confidence intervals and significance levels. For microsimulation model parameters, this is less straight forward. Because the analysis stacks several models, of lung cancer carcinogenesis, lung cancer natural history, lung cancer detectability, and lung cancer mortality (as demonstrated in Figure 1 of the main text), the quantification of the uncertainty by traditional statistics becomes infeasible. The statistical likelihood of one parameter relative to the calibration targets, could depend on the value of another parameter in a different part of the model. Their interaction may not be analytically derived, therefore hampering the calculation of a joint distribution of the parameters. Methods such as Bayesian calibration have been proposed to derive posterior joint distributions of the parameter set conditional on the calibration targets, but these are known to be computationally burdensome, and exceed the possibilities of our implementation, where many parameters are involved.

To study the uncertainty of the parameter estimates, we may follow previous studies that calibrated microsimulation models in a similar manner, and build a range of feasible values of the parameter estimates, holding all other parameters equal. We should note that this does not constitute a confidence interval in the traditional sense, because it does not incorporate the joint distribution of model parameters.

To study parameter uncertainty, we generate a likelihood profile of the parameter set when values are shifted above and below the maximum-likelihood estimates. Similar to a likelihood-ratio test, the 95% range of feasible values is then taken to be the point where the log-likelihood difference with the maximum likelihood exceeds the 97.5 percentile of a Chi-Squared distribution with 1 degree of freedom (critical value 5.024). This reports the 95% range of feasible values, conditional on all other parameters in the model remaining equal. The figure below shows an example of this for the sojourn time of stag IA Adenocarcinoma, which we scale between 0.3 and 1.9 of the maximum likelihood estimate. The range of feasible values is obtained by the point where the log likelihood increases by more than the 97.5th percentile of a 1df Chi-Squared distribution.

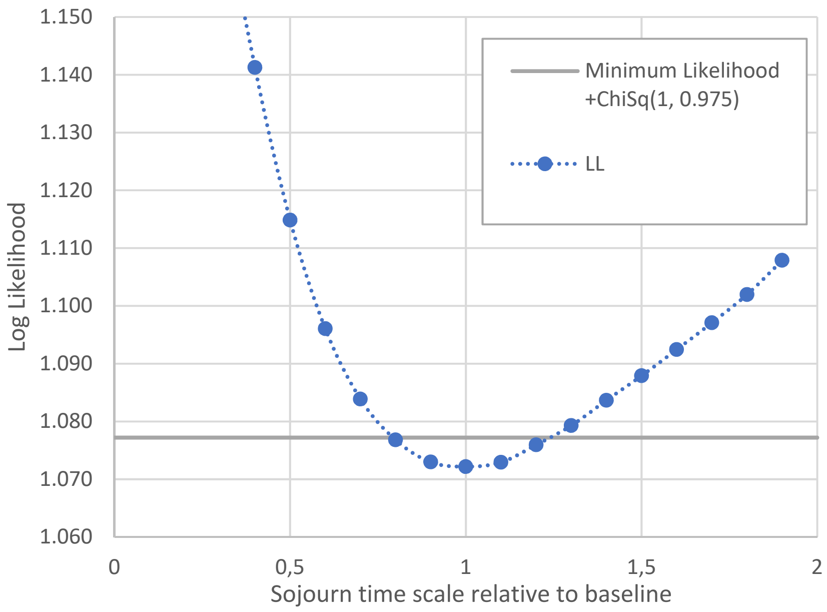

The likelihood profile obtained by scaling the stage IA sojourn time around the point estimate

First we study the uncertainty of the CT sensitivity estimates by jointly shifting the estimated sensitivities by a given number of percentage points up and down relative to their maximum likelihood values. We then interpolate at which value the difference in the likelihood equals the critical value of

the Chi-Squared(1) distribution. We repeat the analysis for 3 runs of the MISCAN model to account for simulation noise, and report the resulting range of feasible values in Supplementary Table 11. We find an uncertainty range of (-5.9%, 5.8%) for the CT sensitivity estimates relative to the point estimates.
Second, we study the uncertainty of the re-estimated preclinical sojourn time for IA Adenocarcinoma, by lengthening and shortening the estimated IA Adenocarcinoma dwell time by a range of values, and interpolating for which factors the likelihood difference passes the critical value of the chi-squared distribution. We find an uncertainty range of (3.08,4.11) years for males and (4.12,5.51) years for females.

Finally, we raise and lower the estimated mortality reduction parameters reported in Figure 4 of the main text and report the range of feasible values per the same methodology. We find a range of feasible values for the mortality reduction parameters of (-9.2%,2,9%).

Supplementary Tables

Supplementary Table 1 – Descriptive Statistics of Dutch-Center NELSON Trial Participants.

		Screening Arm	Control Arm
Age	<55	2000(26.9%)	1906(25.7%)
	55-59	2521(33.9%)	2524(34.0%)
	60-64	1664(22.4%)	1721(23.2%)
	65-69	833(11.2%)	867(11.7%)
	70-74	370(5.0%)	361(4.9%)
	>75	45(0.6%)	42(0.6%)
Smoking Duration	0-30y	783(10.6%)	854(11.5%)
	30-39y	4254(57.5%)	4333(58.5%)
	40-49y	2237(30.2%)	2097(28.3%)
	50y+	128(1.7%)	128(1.7%)
Years since Quit Smoking	Current Smoker	4125(55.6%)	4064(54.7%)
	0-15y	548(7.4%)	559(7.5%)
	15-30y	2673(36.0%)	2734(36.8%)
	30y+	47(0.6%)	40(0.5%)
Cigarettes per Day	0-15	1693(22.9%)	1638(22.1%)
	15-29	4819(65.1%)	4900(66.1%)
	30+	892(12.1%)	876(11.8%)
Packyears	0-20	32(0.4%)	25(0.3%)
	20-30	2255(30.4%)	2226(30.0%)
	30-40	2110(28.4%)	2209(29.7%)
	40+	3009(40.5%)	2958(39.8%)
Sex	Male	6198(83.7%)	6239(84.2%)
	Female	1204(16.3%)	1171(15.8%)
Lung Cancer	All	469(6.3%)	441(6.0%)
	Screen-Detected	229(3.1%)	
	Clinically Detected	240(3.2%)	441(6.0%)

Supplementary Table 1 reports baseline characteristics of participants in the NELSON trial from Dutch screening centers (n=14,856), excluding Belgium participants (n=933) due to data availability.

Supplementary Table 2 - Two Stage Clonal Expansion model parameters by sex, as recalibrated to Dutch population lung cancer incidence.

		Parameter Estimate	
	**Parameter Description**	**Male**	**Female**
**Fixed Parameters**	Stem cell population X	1E8	
	Initiated cells' division rate α	3	
	Gamma-distributed lag time mean	0.0	
**Background rates**	Initiation rate μ_0	8.39E-08*	7.13E-09*
	Malignant Conversion rate μ_1	1.04E-07*	1.25E-07*
	Gamma-distributed lag time sd	0.0	
**Tobacco coefficients**	Tobacco promotion rate coefficient g_c	0.112	0.221*
	Tobacco promotion rate power g_e	0.517	0.516*
	Tobacco malignant-conversion coefficient μ_{1c}	0.534	0.936*
	Tobacco malignant-conversion power μ_{1e}	0.468	0.549*

*: Parameters recalibrated to match Dutch lung cancer incidence with health survey smoking behavior for the period 2000-2020. Original parameter values and the statistical specification of the Two Stage Clonal Expansion model can be found in the literature. [13]

Supplementary Table 3 - MISCAN-Lung histology distribution by sex (in %), as calibrated to population lung cancer incidence and outcomes from the NELSON trial.

	Histology Distribution	
	**Male**	**Female**
**Squamous-cell carcinoma**	28.6	14.3
**Adenocarcinoma**	42.7	56.1
**Small-cell lung cancer**	13.1	16.1
**Other NSCLC**	15.6	13.4

MISCAN-Lung estimated histology distribution of lung cancers, as calibrated to outcomes from the NELSON trial and population level incidence by histology. The distribution by sex represents the share of each histology in all lung cancers, including those not associated with a clinical outcome in the model.

Supplementary Table 4 – Stage and histology distribution of cancers among the Dutch-center participants of the NELSON trial, stratified by trial arm and avenue of diagnosis..

	Histology Distribution (%)		
	Control Arm	Screening Arm	
	Clinically Detected	Clinically Detected	Screen-Detected
Histological Type			
**Squamous-cell carcinoma**	58.3	24.3	17.4
**Adenocarcinoma**	44.0	22.3	33.7
**Small-cell lung cancer**	52.0	37.8	10.2
**Other NSCLC**	43.7	32.5	23.8
TNM Stage			
**1A**	7.3	8.3	51.1
**1B**	7.0	6.3	11.4
**2**	8.6	5.0	9.2
**3A**	12.0	11.7	9.2
**3B**	11.6	10.0	5.2
**4**	41.0	43.8	7.9
**X**	12.5	15.0	6.1

The table reports the share of each histology and TNM stage among NELSON cancers, particularly those detected among Dutch-center participants (n=910). Shares are reported as a percentage of total cancers for clinically detected cancers in the control arm(n=441), among cancers clinically detected in the screening arm (n=240), and screen-detected cancers (n=229). Stage X cancers, those with an unspecified stage at detection are included here, but the stage of cancer is imputed for these cancers before incorporation in the MISCAN model calibration, per methods reported in the supplement.

Supplementary Table 5 - MISCAN-Lung transition probabilities by stage and histology

From	To	Adenocarcinoma	Squamous cell carcinoma	Small cell carcinoma	Other non-small cell carcinomas
Preclinical IA	Preclinical IB	0.85	0.87	0.97	0.92
	Clinical detection IA	0.15	0.13	0.03	0.08
Preclinical IB	Preclinical II	0.88	0.85	0.97	0.94
	Clinical detection IB	0.12	0.15	0.03	0.06
Preclinical II	Preclinical IIIA	0.93	0.87	0.97	0.95
	Clinical detection II	0.07	0.13	0.03	0.05
Preclinical IIIA	Preclinical IIIB	0.87	0.81	0.89	0.87
	Clinical detection IIIA	0.13	0.19	0.11	0.13
Preclinical IIIB	Preclinical IV	0.76	0.65	0.80	0.80
	Clinical detection IIIB	0.24	0.35	0.20	0.20
Preclinical IV	Clinical detection IV	1.00	1.00	1.00	1.00

Parameters were estimated by model calibration to individual-level data of the NLST, the PLCO and the NELSON trial.
*: Updated after calibration to Dutch population-level lung cancer outcomes and NELSON trial individual-level outcomes, per the calibration methods presented further on.

Supplementary Table 6 – MISCAN-Lung sojourn times (mean of Weibull distribution) by stage and histology, after recalibration to NELSON

	Males	Females
**Adenocarcinoma**		
IA	3.56	4.77
IB	0.64	0.86
II	0.46	0.62
IIIA	0.46	0.62
IIIB	0.36	0.48
IV	0.74	0.99
**Other NSCLC**		
IA	1.96	2.31
IB	0.69	0.81
II	0.50	0.59
IIIA	0.50	0.59
IIIB	0.39	0.45
IV	0.80	0.94
**Small-cell lung cancer**		
IA	1.25	1.36
IB	0.44	0.48
II	0.32	0.34
IIIA	0.32	0.35
IIIB	0.25	0.27
IV	0.51	0.55
**Squamous-cell carcinoma**		
IA	2.16	2.15
IB	0.76	0.76
II	0.55	0.55
IIIA	0.55	0.55
IIIB	0.42	0.42
IV	0.88	0.88

Only IA Adenocarcinoma sojourn distribution was recalibrated to NELSON, with other histological types left to NLST-derived values after no improvement from their recalibration was found in the fit to NELSON. Sojourn times are drawn from a Weibull distribution, which takes both mean and shape parameters. Shape is 0.35 for stage IA Adenocarcinoma, 1.44 otherwise.

Supplementary Table 7– Relative cumulative survival (in %, per the Ederer-II method) of lung cancer in the Netherlands for individuals diagnosed 2004-2012, by sex, histology and stage.

	**Cumulative survival (in %) after given period from diagnosis**							
	**Males**				**Females**			
	**1y**	**2y**	**5y**	**10y**	**1y**	**2y**	**5y**	**10y**
**Adenocarcinoma**								
IA	90.6	81.0	61.3	40.9	94.0	86.5	70.0	52.8
IB	77.2	62.8	43.7	28.9	81.9	69.4	50.4	38.7
II	70.9	54.1	36.5	25.4	79.1	63.4	42.9	29.2
IIIA	56.4	33.4	16.4	9.6	64.1	42.7	22.2	13.3
IIIB	42.6	22.4	10.2	5.8	46.3	24.9	11.0	6.5
IV	17.3	5.2	1.4	0.7	23.3	8.7	2.6	1.4
**Other NSCLC**								
IA	91.1	82.5	61.3	40.6	92.6	85.1	67.9	50.9
IB	78.3	64.4	43.9	28.6	81.9	70.1	53.2	37.1
II	72.4	54.7	36.4	26.8	76.6	62.4	44.4	33.5
IIIA	56.7	33.6	14.8	9.1	64.7	41.5	21.5	13.0
IIIB	40.8	19.9	8.1	4.5	44.5	24.5	11.0	7.2
IV	17.1	5.2	1.3	0.7	23.7	8.6	2.1	1.1
**Small-cell lung cancer**								
IA	90.0	80.2	58.7	39.3	92.8	85.5	66.4	51.2
IB	74.7	62.0	44.7	32.1	87.1	77.5	60.7	43.1
II	72.4	53.6	32.9	22.7	71.8	55.1	37.3	24.8
IIIA	57.7	35.0	16.3	11.0	62.7	41.9	22.5	13.7
IIIB	42.9	21.9	8.6	4.8	46.1	26.7	12.3	6.7
IV	16.7	5.1	1.0	0.5	24.0	9.5	2.8	1.6
**Squamous-cell carcinoma**								
IA	90.3	80.0	57.9	38.8	93.5	87.6	72.2	53.2
IB	76.6	61.9	42.7	31.6	83.3	73.1	55.4	40.0
II	70.6	53.4	36.2	24.8	74.8	58.1	38.7	26.1
IIIA	56.4	33.0	15.9	9.6	62.3	41.1	21.6	13.2
IIIB	41.8	21.5	9.8	6.0	47.0	26.4	13.1	8.3
IV	17.1	5.1	1.2	0.6	23.2	8.6	2.2	1.3

Supplementary Table 7 reports survival for lung cancer in the Netherlands for the period contemporary to the NELSON trial, by stage and histology. All-cause survival from the day of incidence is reported for each incident lung cancer case by the Dutch cancer registry. Life tables by single-year birth cohort and sex are taken from Statistics Netherlands [14] to calculate relative survival per the Ederer-II method [15] to isolate cause-specific survival. The MISCAN-Lung model uses Poisson interpolations of the shown survival probabilities to simulate lung cancer survival from the moment of incidence onwards.

Supplementary Table 8 – Midpoints of smoking initiation, smoking cessation and cigarettes per day by self-reported category.

Cigarettes Per Day (CPD) Midpoints										
**Category (CPD)**	<5	5-10	11-15	16-20	21-25	26-30	31-40	41-50	51-60	>60
**midpoint**	3	7.5	13	18	23	28	33	43	53	65

Smoking Initiation Midpoints								
**Category (age)**	<10	10-14	15-19	20-24	25-29	30-34	35-39	>40
**Midpoint**	9	12	17	22	27	32	37	42

Smoking Cessation Midpoints											
**Category (years)**	1/12-5	6-10	11-15	16-20	21-25	26-30	31-35	36-40	41-45	46-50	>50
**Midpoint**	3	8	13	18	23	28	33	38	43	48	52

Supplementary Table 9 -MISCAN-Lung calibration targets for recalibration to individual-level outcomes of the NELSON trial

Calibration Target	Aggregation Level	Assumed Distribution	n-value
Dutch Population incidence rate	5y Cohort, Sex, 5y Age group	Poisson	Lifeyears at risk for lung cancer incidence
Dutch Population histology distribution	Sex, Histology, Stage	Multinomial	Number of cancers
Control arm incidence rate	Sex, Age group, Integer year after baseline	Poisson	Lifeyears at risk for lung cancer incidence
Control arm histology distribution	Sex, Age group, Cancer Histology, Cancer stage	Multinomial	Number of cancers
Screening arm clinical incidence rate	Sex, Age group, Integer year after baseline	Poisson	Lifeyears at risk for lung cancer incidence
Screening arm screen-detected incidence rate	Sex, Age group, Integer year after baseline	Poisson	Lifeyears at risk for lung cancer incidence
Screening arm histology distribution of clinically detected cancers	Sex, Age group, Cancer stage	Multinomial	Clinically detected cancers
Screening arm histology distribution of screen-detected cancers	Sex, Age group, Cancer stage, Round of detection	Multinomial	Screen-detected cancers
Control arm mortality rate of clinically detected cancers	Sex, Cancer stage	Poisson	Lifeyears at risk for lung cancer mortality
Screening arm mortality rate of screen detected cancers	Sex, Cancer stage	Poisson	Lifeyears at risk for lung cancer mortality
Screening arm mortality rate of clinically detected cancers	Sex, Cancer stage	Poisson	Lifeyears at risk for lung cancer mortality

Supplementary Table 10- Parameters recalibrated to NELSON individual-level outcomes

MISCAN-Lung Model Parameter	Dimension	Bounded in calibration
CT sensitivity coefficient $\alpha_{s,h}$, for stage s and histology h	Stage (6), Histology (4)	Minimally 0 for any $s \neq IA$
Repeat screen increment coefficient in sensitivity relative to baseline β_h	Histology (4)	Minimally 0
Clinical detection rate stage IA cancers	Histology (4)	[0, 1]
Clinical detection rate stage IB cancers	Histology (4)	To exceed the IA detection rate and not to exceed 1.0
Weibull preclinical duration distribution mean and shape	Histology (4)	Within 0.5 and 2.0 of the starting value
Share of cancers by histology	Histology (4), Sex (2)	To sum up to 1.0 for each sex
Two-Stage Clonal Expansion Model - Background rates:<br>Initiation rate, Malignant Conversion rate	Sex (2)	Confidence bounds per Meza et al.*
Two-Stage Clonal Expansion Model - Tobacco Dose-Response coefficients:<br>Promotion rate, promotion power, malignant conversion rate, malignant conversion power	Only for Females	Confidence bounds per Meza et al.*

**: Bounds expanded for some parameters when converging to edges of Meza et al. confidence bounds.*

Supplementary Table 11 – Profile likelihood range of feasible CT sensitivity values

CT Sensitivity Uncertainty Estimates		
	**NELSON baseline screen (%)**	**NELSON repeat screen (%)**
**Adenocarcinoma**		
IA	41.0(32.1-47.2)	70.9(62.0-77.1)
IB	49.0(40.2-55.2)	77.1(68.3-83.3)
II	56.3(47.4-62.5)	81.9(73.0-88.0)
IIIA	99.3(90.4-100)	99.3(90.4-100)
IIIB	99.5(90.6-100)	99.8(90.9-100)
IV	99.9(91.0-100)	99.9(91.1-100)
**Squamous-cell Carcinoma**‡		
IA	30.1(21.2-36.3)	30.1(21.2-36.3)
IB	30.4(21.5-36.5)	30.4(21.5-36.5)
II	79.3(70.5-85.5)	79.3(70.5-85.5)
IIIA	96.7(87.8-100)	96.7(87.8-100)
IIIB	99.3(90.4-100)	99.3(90.4-100)
IV	99.9(91.0-100)	99.9(91.0-100)
**Other NSCLC**‡		
IA	24.6(15.7-30.7)	24.6(15.7-30.7)
IB	26.4(17.5-32.5)	26.4(17.5-32.5)
II	39.6(30.8-45.8)	39.6(30.8-45.8)
IIIA	67.5(58.6-73.6)	67.5(58.6-73.6)
IIIB	89.8(80.9-95.9)	89.8(80.9-95.9)
IV	99.2(90.3-100)	99.2(90.3-100)
**Small-cell Lung Cancer**‡		
IA†		
IB†		
II†		
IIIA	78.7(69.8-84.8)	78.7(69.8-84.8)
IIIB	96.8(87.9-100)	96.8(87.9-100)
IV	99.9(91.0-100)	99.9(91.0-100)

Sojourn Time Uncertainty Estimate	
IA Adenocarcinoma – Males	3.56(3.08-4.11)
IA Adenocarcinoma - Females	4.77(4.12-5.51)

Mortality Reduction Parameter Uncertainty Estimate	
IA	83.1(74-86.1)
IB	83.1(73.9-86.1)
II	48.4(39.2-51.4)
IIIA	26.7(17.6-29.7)
IIIB	26.7(17.5-29.7)
IV	4.6(0-7.6)

Supplementary Table 11 reports results of the profile likelihood analysis, exploring the statistical uncertainty surrounding the point estimates presented in the main text. The point estimates of NELSON-calibrated parameters are shown, together with the 95% range of feasible values obtained by varying the sensitivities, the sojourn times and the mortality reduction parameters in turn, and observing where the likelihood difference with the maximum likelihood estimate exceeds 2.5 and 97.5th percentiles of the Chi-Squared distribution.

†: Not recalibrated for the MISCAN-Lung NELSON calibration; the stage-histology combination was not observed in NELSON.

‡: Estimated $\beta_h = 0$, i.e. no differences found between repeat and baseline screen sensitivity for squamous-cell, small-cell and other NSCLC cancers.

Supplementary Figures

Supplementary Figure 1 - Observed vs MISCAN-Lung simulated incidence rate of all lung cancer in the Dutch population of NELSON-eligible ages for the period 2000-2020.

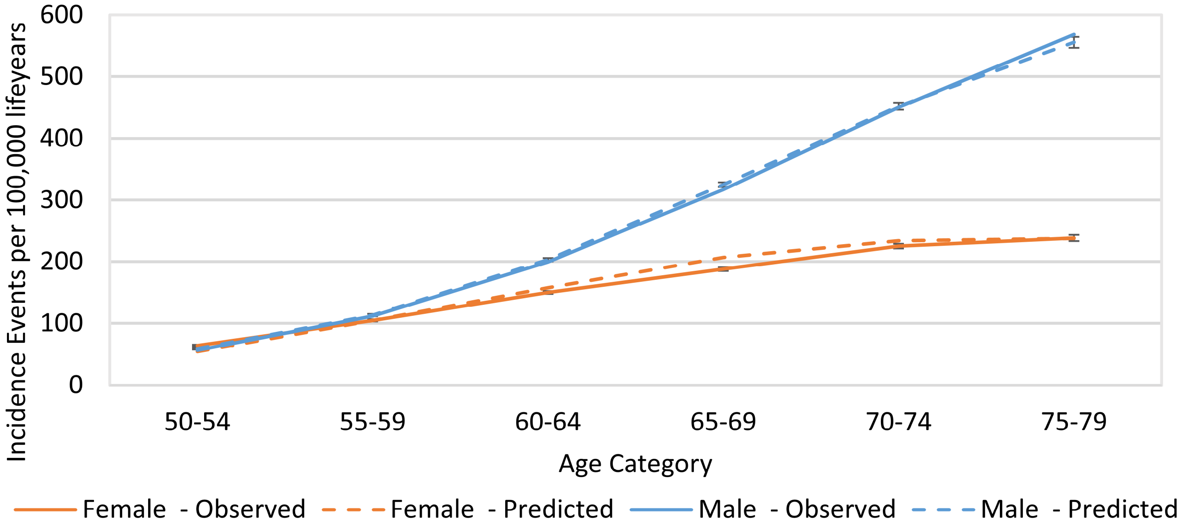

The MISCAN-Lung simulated and observed population-level incidence of lung cancer per 100,000 life-years in the period 2000-2020 for the Netherlands. Lung cancer events are taken from the Netherlands Cancer Registry, life-years lived are informed by population sizes by year, age and sex taken from Statistics Netherlands. The MISCAN-Lung estimates of lung cancer incidence are generated per the methods described in supplement A, accounting for cohort- and sex-specific smoking behavior in the lifetimes preceding lung cancer incidence.

Supplementary Figure 2 - MISCAN-Lung predicted number of deaths among screen-detected cases vs observed number in NELSON, by stage at detection.

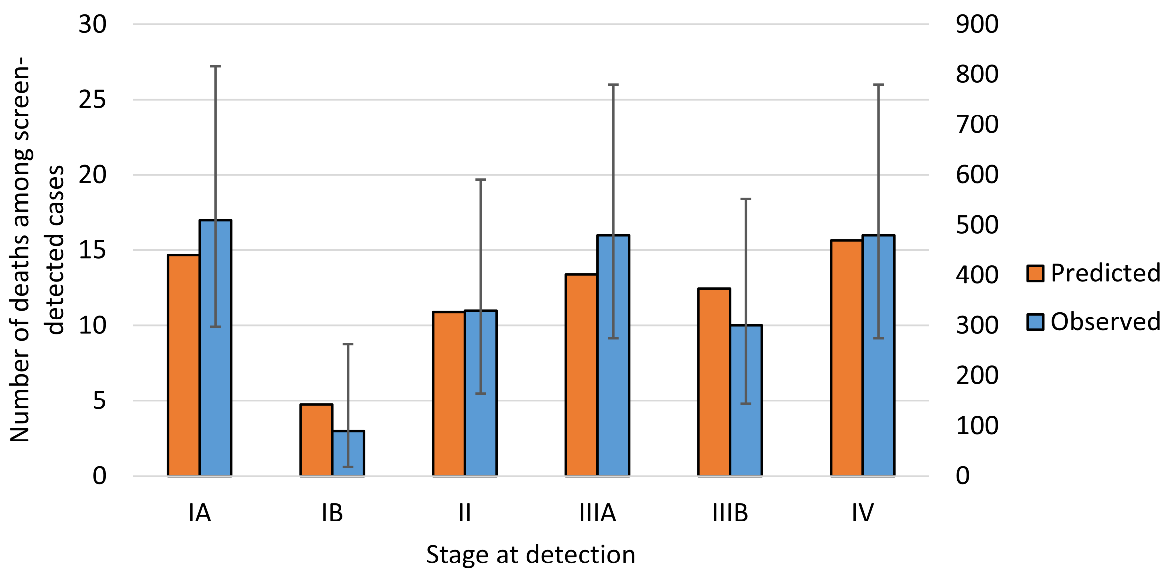

Note: 95% Confidence intervals assume a Poisson mortality rate.

Supplementary Figure 3 - MISCAN-Lung predicted number of interval cancers in the NELSON screening arm vs observed number in NELSON, by stage at detection and screening interval

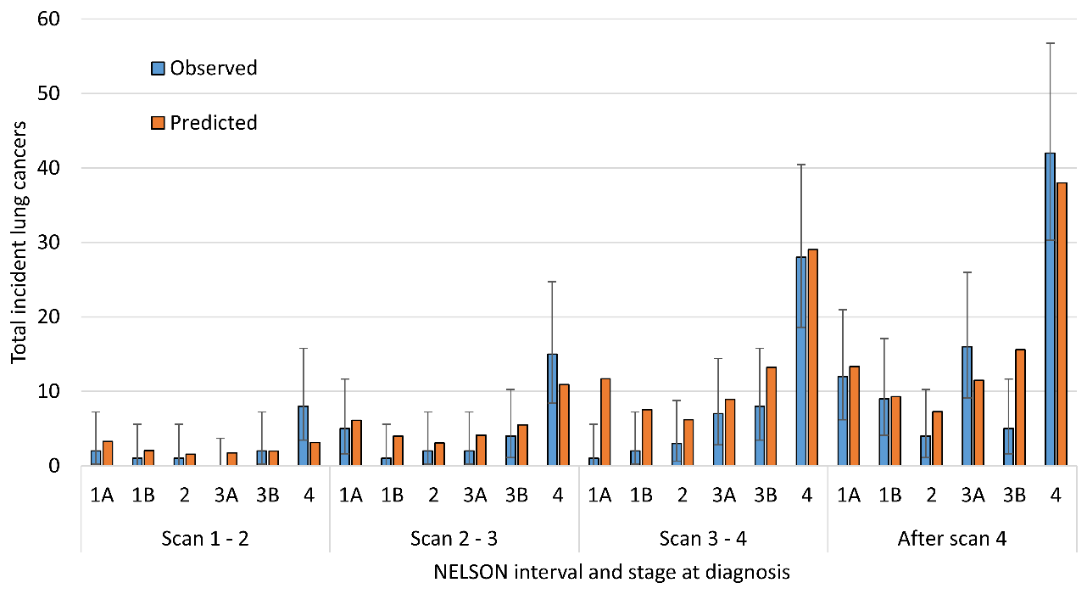

95% Confidence intervals assume a poisson incidence rate specific to the stage of the cancer and the screening interval.

Supplementary Figure 4 - MISCAN-Lung simulated lung cancers by age and sex for the period 2000-2020 in the Netherlands.

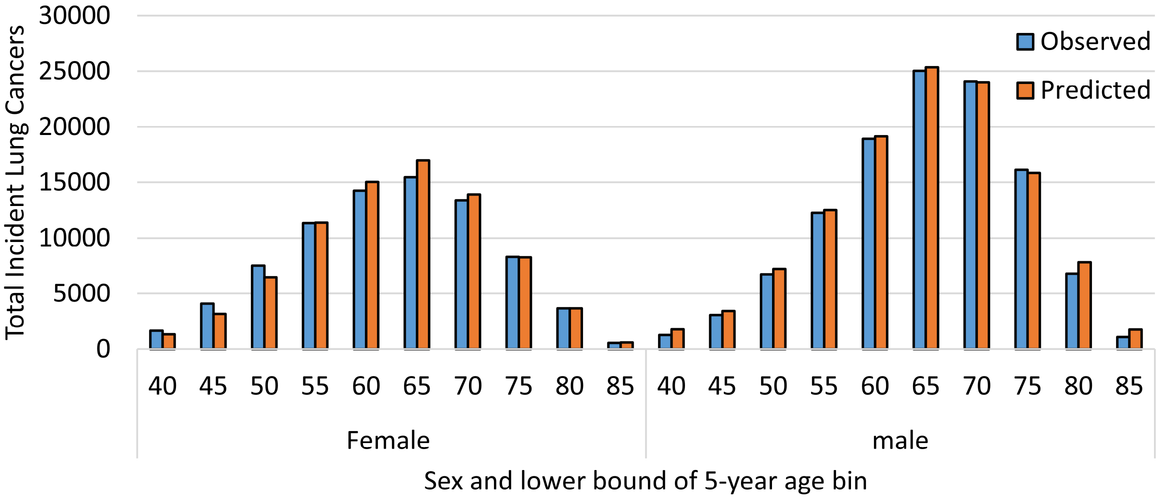

Note: Supplementary Figure 4 reports MISCAN-Lung predicted gross lung cancer incidence for the period 2000-2020 for the country of the Netherlands, summed across males and females. Dutch health survey data on smoking behaviour by sex for the 1930 to 1979 cohorts is used to inform the MISCAN-Lung microsimulation of life histories. Cohort sizes are based on the 2020 population composition of the Netherlands.

Supplementary Figure 5 Lung cancer progression in the MISCAN-Lung model

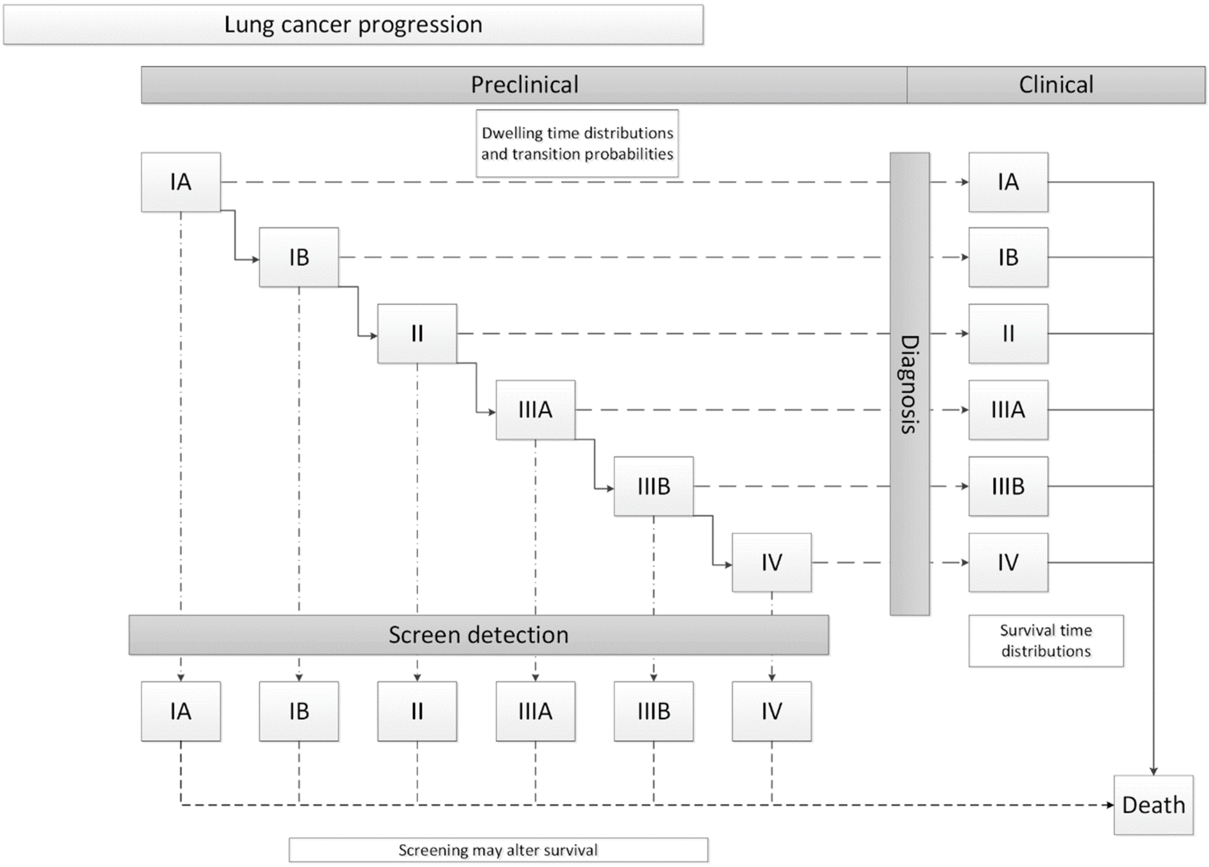

Note: Once lung cancer has developed, it will progress from less advanced to more advanced preclinical stages until it is clinically detected. This process is similar for all histologies, however, the average time spent in the current state differs by histology, preclinical cancer stage and gender. The probability that a cancer progresses to a more advanced preclinical stage or is diagnosed clinically (e.g., diagnosed due to symptoms) is modelled by histology and stage. Screening may detect cancers in each of the preclinical screen-detectable states, depending on the sensitivity of the screening test for the specific histology and preclinical detectable state. Upon detection of lung cancer by screening, a person's life history may be altered. Detection by screening may prevent the lung cancer death, allowing them to resume their normal (lung cancer free) life history. The probability of mortality prevention differs by the stage of detection. After clinical detection or screen detection (without successful mortality prevention) the patient's duration of survival follows a histology and stage specific survival function, which is piecewise uniformly distributed. A person may also die from causes other than lung cancer.

Supplementary Figure 6 - MISCAN-Smoking History Generator predictions of current smoking prevalence compared to observed Dutch Health Survey smoking prevalence for Male 10-year cohorts 1930-1970

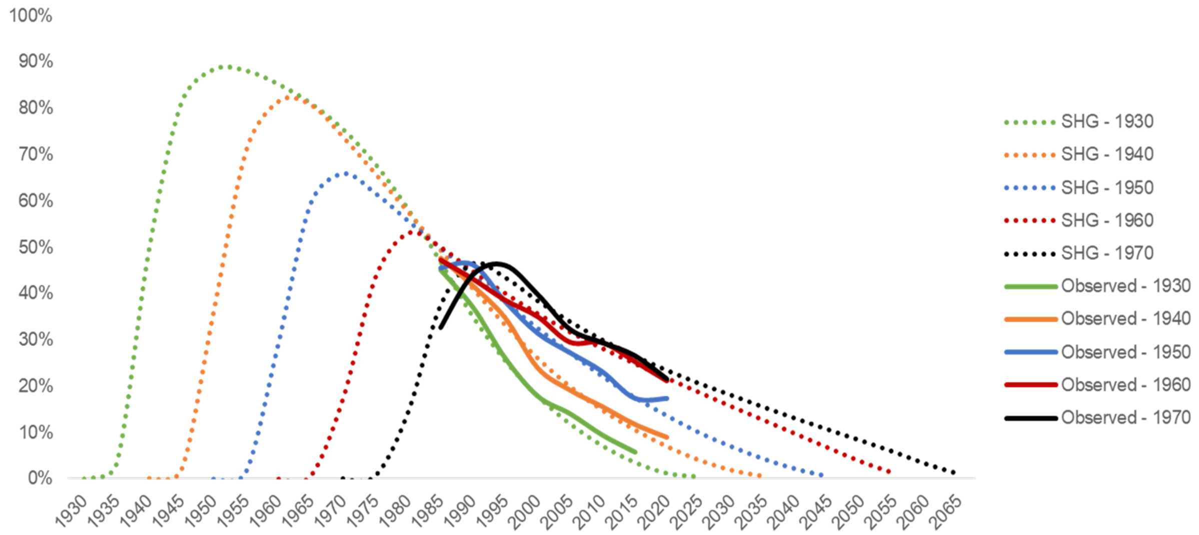

Supplementary Figure 7 - MISCAN-Smoking History Generator predictions of current smoking prevalence compared to observed Dutch Health Survey smoking prevalence for Female 10-year cohorts 1930-1970

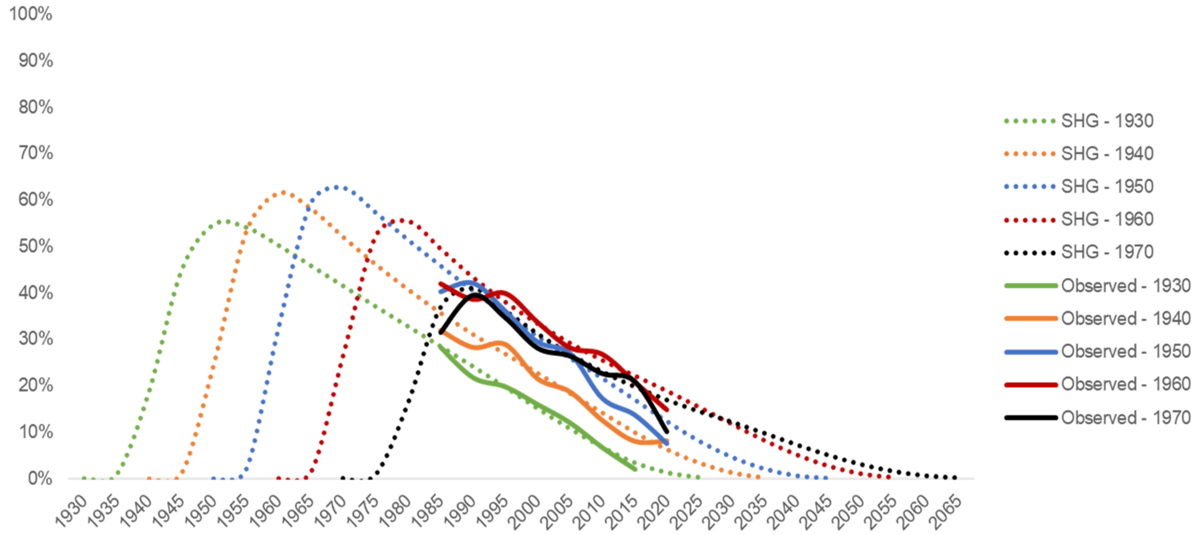

References

1. Tomonaga Y, Ten Haaf K, Frauenfelder T, et al. Cost-effectiveness of low-dose CT screening for lung cancer in a European country with high prevalence of smoking-A modelling study. *Lung cancer (Amsterdam, Netherlands)* 2018;121:61-69.
2. ten Haaf K, van Rosmalen J, de Koning HJ. Lung Cancer Detectability by Test, Histology, Stage, and Gender: Estimates from the NLST and the PLCO Trials. *Cancer Epidemiology Biomarkers & Prevention* 2015;24:154.
3. Meza R, ten Haaf K, Kong CY, et al. Comparative analysis of 5 lung cancer natural history and screening models that reproduce outcomes of the NLST and PLCO trials. *Cancer* 2014;120:1713-1724.
4. Heidenreich WF, Luebeck EG, Moolgavkar SH. Some properties of the hazard function of the two-mutation clonal expansion model. *Risk Anal* 1997;17:391-399.
5. Meza R, Hazelton WD, Colditz GA, Moolgavkar SH. Analysis of lung cancer incidence in the nurses' health and the health professionals' follow-up studies using a multistage carcinogenesis model. *Cancer Causes & Control* 2008;19:317-328.
6. Pérez-Stable EJ, Marín BV, Marín G, et al. Apparent underreporting of cigarette consumption among Mexican American smokers. *American Journal of Public Health* 1990;80:1057-1061.
7. Gallus S, Tramacere I, Boffetta P, et al. Temporal changes of under-reporting of cigarette consumption in population-based studies. *Tobacco Control* 2011;20:34-39.
8. Liber AC, Warner KE. Has Underreporting of Cigarette Consumption Changed Over Time? Estimates Derived From US National Health Surveillance Systems Between 1965 and 2015. *Am J Epidemiol* 2018;187:113-119.
9. Xu DM, Gietema H, de Koning H, et al. Nodule management protocol of the NELSON randomised lung cancer screening trial. *Lung Cancer* 2006;54:177-184.
10. de Koning HJ, van der Aalst CM, de Jong PA, et al. Reduced Lung-Cancer Mortality with Volume CT Screening in a Randomized Trial. *N Engl J Med* 2020;382:503-513.
11. Mehta HJ, Ravenel JG, Shaftman SR, et al. The utility of nodule volume in the context of malignancy prediction for small pulmonary nodules. *Chest* 2014;145:464-472.
12. Han D, Heuvelmans MA, Oudkerk M. Volume versus diameter assessment of small pulmonary nodules in CT lung cancer screening. *Transl Lung Cancer Res* 2017;6:52-61.
13. Meza R, Hazelton WD, Colditz GA, Moolgavkar SH. Analysis of lung cancer incidence in the Nurses' Health and the Health Professionals' Follow-Up Studies using a multistage carcinogenesis model. *Cancer Causes Control* 2008;19:317-328.
14. Life expectancy by sex and cohort. Statistics Netherlands; 2000-2020 Available at https://opendata.cbs.nl/statline/#/CBS/nl/dataset/80333ned/table?dl=72252.
15. Dickman PWC, Enzo. Estimating and modeling relative survival. *The Stata Journal, StataCorp LP* 2015;15:186-215.
